# Occupational stress and associated factors among nurses working at public hospitals of Addis Ababa, Ethiopia, 2022; A hospital based cross-sectional study

**DOI:** 10.3389/fpubh.2023.1147086

**Published:** 2023-04-18

**Authors:** Elshaday Bekele Werke, Zewdu Shewangizaw Weret

**Affiliations:** ^1^Department of Public Health, School of Public Health, College of Medicine and Health Science, Dilla University, Dilla, Ethiopia; ^2^Department of Psychiatry, Menelk II Medical and Health Science College, Kotebe University of Education, Addis Ababa, Ethiopia

**Keywords:** occupational stress, nurses, public hospitals, Addis Ababa, Ethiopia

## Abstract

**Background:**

By its very nature, the nursing profession involves a lot of stress. Working in this field includes interacting with individuals who are already under a great deal of stress. Workplace stress affects the quality of services provided and also causes staff burnout, departure, and absenteeism.

**Objective:**

This study is to determine occupational stress and associated factors among nurses working at public hospitals, Addis Ababa, Ethiopia, 2022.

**Materials and methods:**

An institutional based cross sectional study was conducted among 422 nurses working at public hospitals from March 1 to April 1/2022. Simple random sampling technique was used to select public hospitals. The calculated sample size was allocated proportionally to each hospital based on the number of nurses. Finally, systematic sampling method was used to approach the study participants. The data was collected by using a self-administered structured questionnaire (Expanded Nursing Stress Scale). The collected data was entered by Epi-data version 3.1 and analyzed by SPSS version 23. Descriptive analysis such as frequency distribution and measure of central tendency and variability (mean and standard deviation) was computed to describe variables of the study. Binary logistic regression was used to assess associations between dependent and independent variables. The degree of associations was interpreted using odds ratio (OR) and 95% confidence interval (CI) and statically significance at value of *p* < 0.05. The result was presented using text, tables, and graphs.

**Result:**

The study finding showed that 198 (47.8%) of nurses were occupationally stressful. Factors significantly associated with occupational stress among nurses were having children (no: AOR = 0.46, 95% CI: 0.22, 0.96) and work shift (rotating: AOR = 2.89, 95% CI: 1.87, 4.45).

**Conclusion:**

In this study, job stress affected over half of the nurses. The presence of children and respondents’ work shifts were personal characteristics that were significantly linked to job stress. Therefore based on this result the government policy makers, different stakeholders and hospitals need to collaborate to reduce nurses job related stress.

## 1. Introduction

A common way to define stress is as a feeling of being overburdened, wound up tight, tense, and concerned (1). It is a disruptive condition that develops in reaction to harmful effects from the internal or external settings (2).

Occupational stress is defined by the National Institute for Occupational Safety and Health (NIOSH) as “the negative physical and emotional reactions that occur when the requirements of the job do not match the worker’s talents, resources, or needs” (3). Occupational stress is sometimes referred to as job stress and/or work-related stress (WRS) in an organizational environment. In organizations, both phrases are frequently used interchangeably, but their meanings are the same (4).

One of the most significant sources of occupational stress is the workplace (5). Stress at work is a topic that psychologists, counselors, and employers are all quite concerned about (6).

In general, nursing is seen as a difficult and stressful career (7). Numerous studies have shown that working in nursing is stressful, which can have an adverse effect on one’s physical and mental health as well as their professional performance (8).

Occupational stress can have a number of detrimental effects on both the individual and the company. Poor physical and mental health as well as organizational costs was documented at the individual and organizational levels (9). Additionally, stress has a cost in terms of one’s health, wellbeing, and job satisfaction. It also has a cost for companies in terms of absenteeism and turnover, which may have an effect on the standard of patient care (10).

In organizations, stress has become a bigger issue over time (11). Stress varies in intensity depending on the circumstance and the person, and if it is not well managed, it can prevent people and organizations from reaching their objectives. The consequences of stress on medical workers, and nurses in particular, have drawn a lot of attention (12). Due to the nature of their occupation and the system in which they operate, nurses are perceived to be under more stress than the majority of individuals (13). The first of 40 stressful professions identified by the National Association of Safety Professionals is nursing (14).

In any healthcare facility, nurses make up the majority of the workforce and play a crucial role in patient care. Nurses provide direct care, and providing care is an interpersonal activity characterized by expert nursing, interpersonal sensitivity, and close connections, as well as by effective communication and the use of professional knowledge and abilities. Nursing staff members are occasionally asked to work lengthy hours without getting enough rest (15, 16). Consequently, the nursing profession is highly stressful. Furthermore, the occupation requires interacting with individuals who are under a great deal of stress themselves. Patients can occasionally be challenging, scared, or angry, and nurses may find themselves responding with an increasing irritation and anger that may result in quitting the profession (17, 18).

The major causes of stress among nurses at work include working shifts, long hours, a lack of control, poor relationships with coworkers, low pay, and unfavorable working conditions (19).

According to research, nurses who experience high levels of job-related stress and physical and mental health issues are more likely to quit their jobs, clash with coworkers, experience intense displacement, suffer from poor health and be unable to complete tasks, exhibit vulnerabilities in professional communication, and, as a result, provide lower-quality care and become dissatisfied with their careers. The influence could also have detrimental effects on patient care, such as medication errors and inadequate care for those receiving it (20).

Depending on research carried out in several nations, stress in particular in the nursing profession has been a significant global issue. A research conducted among staff members of a health authority in the United Kingdom revealed that nurses are under the most pressure out of all medical professionals (21). According to a research done in India, 87.4% of Indian nurses reported feeling stressed (22). Based on a research done in the Greater Accra Region of Ghana, the number of hours that nurses work, their financial situation, and patient deaths are all sources of stress (23).

In comparison to other African nations, Ethiopia has one of the lowest ratios of health personnel to population. One healthcare professional was assigned to every 4,050 people, according to a report from the World Health Organization. Resources shortages, macroeconomic problems, and governmental factors are the causes of Ethiopia’s human resource crisis (24, 25). However, the final report of the Health Sector Development Program (HSDP) IV in Ethiopia indicated that there was a lack of nursing staff on a national level, with a nurse to population ratio of 1:3,870 (26). In Ethiopia, a study conducted in the Jima Zone in the southwest of the country revealed an average total job-related stress level of 58.46 ± 12.62, while in the East Gojjam Zone Public hospitals in the northwest, 57.3% of nurses reported experiencing occupational stress (15, 18).

The recent acceleration in the rate of COVID-19’s spread resulted in a heavy workload, physical exhaustion, a high risk of infection, and ethical conflicts regarding decisions about which patients should be given priority. These factors led to significant psychological stress in healthcare professionals. Given that they spend more time with COVID-19 patients than any other healthcare provider, nurses are disproportionately impacted by the pandemic (27, 28).

The findings of this study will provide information on the prevalence and associated factors of occupational stress to nurses, hospital managers, and health policymakers. It also help health care institutions; particularly hospitals to recognize factors related to stress & help them to take corrective measures in attempt to create conducive environment and to improve the health status of their employees as well as efficiency and quality of care. In addition to this the data gathered will aid in the development of appropriate interventions for stress reduction strategies and programs. Furthermore, this research will create knowledge that may be used as input for future research in the same field.

Although study on the topic of work-related stress among nurses in developed countries has been documented, there is currently little data backed by studies conducted in developing nations, such as Ethiopia. Thus, this study determines the prevalence of stress among nurses in the study area as well as its contributing elements.

## 2. Materials and methods

### 2.1. Study setting and design

This study was conducted in Addis Ababa which is the capital city of Ethiopia. It is located at the center of the country with an estimated area of 527 square kilometers (29). Currently, Addis Ababa has 12 state run and more than 40 private hospitals (29). Five public hospitals namely, Menelik II, St. Paul, St. Peter, Yekatit 12 and Zewditu memorial hospitals was included in this study. The study was done from March 1 to April 1/2022. Institution based cross sectional study was conducted.

### 2.2. Source population and study population

The source population was nurses who were working at public hospitals of Addis Ababa and the study population was nurses who were working at selected public hospitals of Addis Ababa.

### 2.3. Inclusion criteria and exclusion criteria

Nurses who had experience of working for at least 6 months at public hospitals were included whereas nurses who were ill or on leave (annual, maternal, or sick) during data collection period were excluded.

### 2.4. Sample size and sampling technique

A single population proportion calculation was used to get the sample size. The previous research’s 49.2% prevalence rate of occupational stress is used to compute the sample size (30). Five hospitals were selected by simple random sampling technique. The calculated sample size was allocated proportionally to each hospital based on the number of nurses. Finally, simple random sampling method was used to approach the study participants.

### 2.5. Data collection tools

Data was collected using self-administered structured questionnaire. There are three sections in the questionnaire. Socio-demographic information, working place data, and Modified Expanded Nursing Stress Scale make up Parts 1 through 3. An instrument for measuring work-related stress is the Expanded Nursing Stress Scale (31). The ENSS measures occupational stress by using 54 items over eight subscales. The response options on the ENSS questionnaire, which is often created using a likert scale format, typically suggest that stress levels are (1 = never stressful, 2 = occasionally stressful, 3 = frequently stressful, and 4 = always stressful). The responder agreed that the scenario was stressful to a greater extent the higher the score. An overall chronbach’s alpha score of 0.9 indicates that the instrument is reliable (31).

### 2.6. Data collection procedure

Five BSc nurses one for each hospital as data collector and two BSc nurses as supervisor working outside the selected hospitals was recruited. On the day of data collection, the data collectors explained the purpose of the study to the participants before data collection. Then self-administered structure questioner was distributed by giving appropriate instruction to assist respondents how to fill the questions. The study was done from March 1 to April 1/2022.

### 2.7. Data quality control

A pre-test was conducted using 5% of the sample size among nurses working at Ras Desta Damtew hospital 2 weeks prior to data collection and necessary amendment was made. To ensure quality of data training was given for data collectors and supervisors on data collection tool and data collection procedure. Data completeness was checked by data collectors and principal investigator.

### 2.8. Data processing and analysis

Data was coded, cleaned and entered to Epi-data version 3.1 and exported to Statistical Package for Social Science (SPSS) version 23 for analysis. Descriptive analysis such as frequency distribution and measure of central tendency and variability (mean and standard deviation) was computed to describe variables of the study. Binary logistic regression was used to assess associations between dependent and independent variables. The degree of associations was interpreted using odds ratio (OR) and 95% confidence interval (CI) and statically significance at value of *p* < 0.05. The result was presented using text, tables, and graphs.

## 3. Results

### 3.1. Socio demographic characteristics of the respondents

This study had a response rate of 414 (98.1%). In all, 178 (43%) men and 236 (57%) women took part in the study. The respondents’ average age was 27.85 ± 4.28 years. According to this study, 350 (84.5%) of individuals had a BSc degree. Nurses with 5 to 10 years of professional experience made up more than 50% of the participants. Additionally, 179 (43.2%) respondents had children, while 190 (45.9%) respondents were married (Table 1).

**Table 1:** Socio-demographic characteristics of nurses working at public hospitals of Addis Ababa, Ethiopia, 2022 (*N* = 414).

Variables	Categories	Frequency	Percent
Age	<25 years	43	10.4
	25–30 years	217	52.4
	>30 years	154	37.2
Gender	Male	178	43
	Female	236	57
Marital status	Single	220	53.1
	Married	190	45.9
	Divorced	4	0.96
Children	Yes	179	43.2
	No	235	56.8
Level of education	Diploma holder	25	6
	Bachelor degree	350	84.5
	Master degree	39	9.4
Experience	<5 years	105	25.4
	5–10 years	268	64.7
	>10 years	41	9.9
Monthly salary (in birr)	<5,000	35	8.5
	5,000–9,000	326	78.7
	>9,000	53	12.8

### 3.2. Work place characteristics of the respondents

Majority of participants 239 (57.7%) reported working shift of them were rotating. Most of participants 250 (60.4%) had a daily work schedule of no more than 8 h (Table 2).

**Table 2:** Work place characteristics of nurses working at public hospitals of Addis Ababa, Ethiopia, 2022 (*N* = 414).

Variables	Categories	Frequency	Percent
Working unit	Medical ward	116	28.0
	Surgical ward	58	14.0
	Obstetrics and Gynecology	36	8.7
	Pediatrics	67	16.2
	Other	137	33.1
Work shift	Fixed	175	42.3
	Rotating	239	57.7
Hours worked per day	≤8 h	250	60.4
	>8 h	164	39.6

### 3.3. Prevalence of occupational stress among nurses

**Figure 1:**
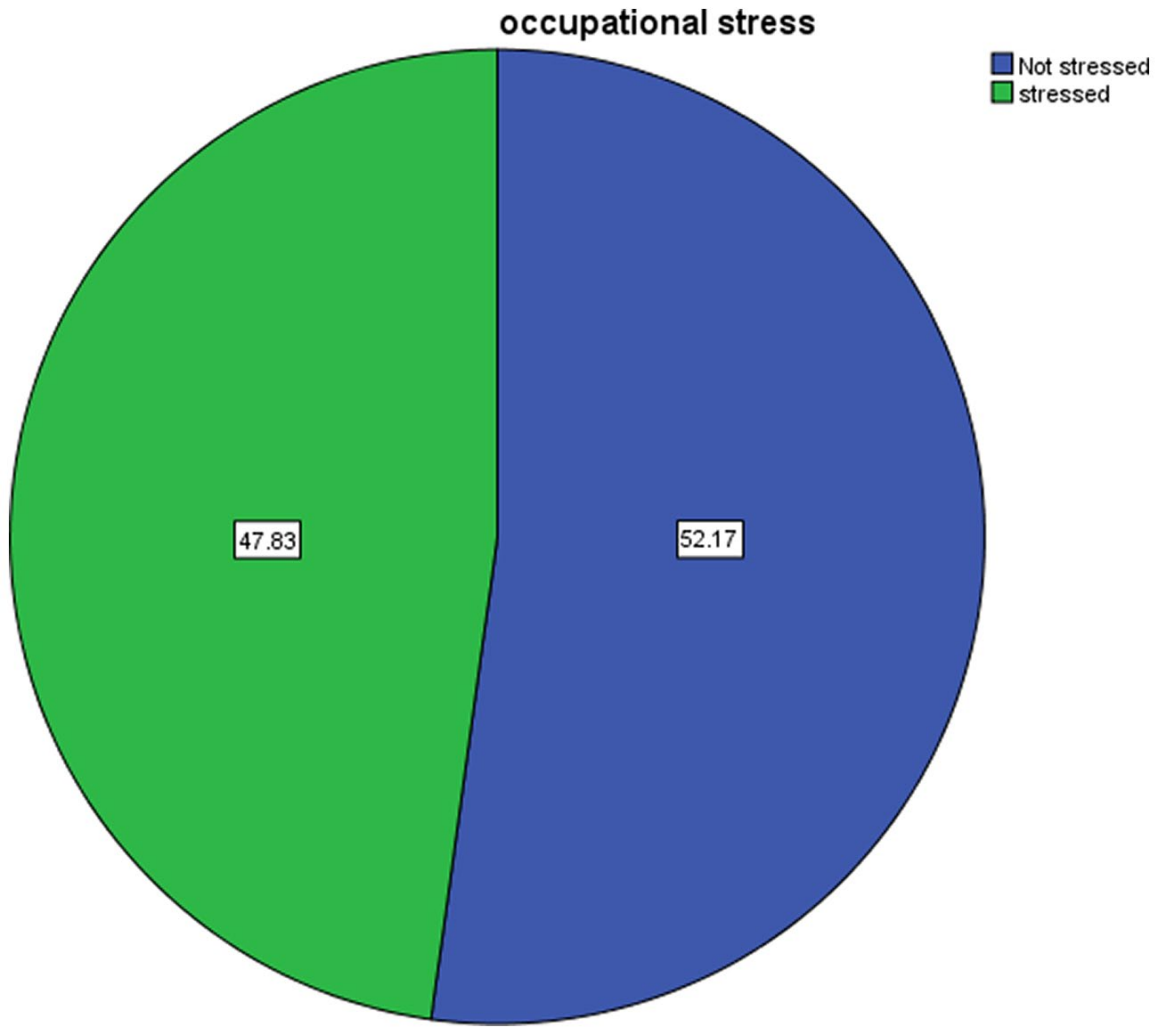
Prevalence of occupational stress among nurses working at Addis Ababa public hospitals, Ethiopia, 2022.

In order to assess the prevalence of occupational stress, participants who scored below the mean value were labeled as “Not stressed,” while those who scored the mean value or higher were categorized as “Stressed.” As a result, 198 (47.8%) of nurses reported having occupational stress (Figure 1).

The mean score of the occupational stress subscales was computed. According to this research, the least stressful aspects of nurses’ jobs were problems with peers while the most stressful aspects were death and dying, uncertainty about treatment, and conflict with physician (Table 3).

**Table 3:** Mean score of response of nurse to ENSS in Addis Ababa public hospitals, Ethiopia, 2022 (*N* = 414).

Subscales	Number of Items	Mean
Psychological factors		
Death and dying	7	2.87
Uncertainty concerning treatment	9	2.53
Inadequate emotional preparation	3	2.36
Physical factors		
Workload	9	2.476
Social factors		
Conflict with physician	5	2.478
Problems with peers	6	2.29
Problems with supervisors	7	2.40
Patient and family	8	2.46

### 3.4. Factors associated with occupational stress

The relationship between the independent variables and the dependent variable was examined using binary logistic regression. The multivariate analysis included all independent variables with *p*-values less than 0.25 in the bivariate analysis, and *p*-values less than 0.05 in multiple logistic regressions were regarded as significant. The results of the multivariate analysis showed that working shifts and having children were both strongly related to job stress.

According to the findings, nurses without children were 54% less anxious than nurses with children (AOR: 0.46, 95% CI: 0.22, 0.96). Working rotating shifts increased respondents’ risk of occupational stress by 2.8 times compared to working fixed shifts (AOR = 2.89, 95% CI: 1.87, 4.45) (Tables 4, 5).

**Table 4:** Socio demographic results of bivariate and multivariate binary logistic regression of factors associated with occupational stress among nurses working at public hospitals of Addis Ababa, Ethiopia, 2022 (*N* = 414).

Variables	Categories	Occupational stress		COR (95% CI)	AOR (95% CI)
		Yes, *N* (%)	No, *N* (%)		
Age	<25 years	23 (53.5%)	20 (46.5%)	1.00	
	25–30 years	106 (48.8%)	111 (51.2%)	0.83 (0.43, 1.6)	
	>30 years	69 (44.8%)	85 (55.2%)	0.70 (0.35,1.39)	
Gender	Male	81 (45.5%)	97 (54.5%)	1.00	
	Female	117 (49.6%)	119 (50.4%)	1.17 (0.79, 1.73)	
Marital status	Single	119 (29.71%)	101 (24.39%)	1.00	1.00
	Married	72 (37.9%)	118 (62.1%)	0.51 (0.34, 0.76)*	0.54 (0.34, 0.84)
	Divorced	2 (50%)	2 (50%)	0.84 (0.11, 6.13)	1.69 (0.21, 13.56)
Children	Yes	78 (43.6%)	101 (56.4%)	1.00	1.00
	No	120 (51.1%)	115 (48.9%)	1.35 (0.91, 1.99)*	0.46 (0.22, 0.96)**
Level of education	Diploma holder	12 (48.0%)	13 (52.0%)	1.00	
	Bachelor degree	163 (46.6%)	187 (53.4%)	0.94 (0.41, 2.12)	
	Master degree	23 (59.0%)	16 (41.0%)	1.55 (0.56, 4.28)	
Experience	<5 years	58 (55.2%)	47 (44.8%)	1.00	1.00
	5–10 years	125 (46.6%)	143 (53.4%)	0.70 (0.45, 1.11)*	0.77 (0.46, 1.28)
	>10 years	15 (36.6%)	26 (63.4%)	0.46 (0.22, 0.98)*	0.89 (0.34, 2.36)
Monthly salary (in birr)	<5,000	17 (48.6%)	18 (51.4%)	1.00	1.00
	5,000–9,000	166 (50.9%)	160 (49.1%)	1.09 (0.54, 2.20)	1.59 (0.74, 3.40)
	>9,000	15 (28.3%)	38 (71.7%)	0.41 (0.17, 1.02)*	0.63 (0.23, 1.73)

CI = confidence interval, COR = crude odds ratio, AOR = adjusted odds ratio.

* *p* < 0.25;

** *p* < 0.05.

**Table 5:** Work place results of bivariate and multivariate binary logistic regression of factors associated with occupational stress among nurses working at public hospitals of Addis Ababa, Ethiopia, 2022 (*N* = 414).

Variables	Categories	Occupational stress		COR (95% CI)	AOR (95% CI)
		Yes, *N* (%)	No, *N* (%)		
Working unit	Medical unit	60 (51.7%)	56 (48.3%)	1.00	1.00
	Surgical unit	28 (48.3%)	30 (51.7%)	0.74 (0.32, 1.72)	0.47 (0.18, 1.21)
	Obstetrics and Gynecology	20 (55.6%)	16 (44.4%)	0.85 (0.40, 1.81)	0.73 (0.31, 1.68)
	Pediatrics	28 (41.8%)	39 (58.2%)	0.57 (0.25, 1.3)*	0.63 (0.24, 1.62)
	Other unit	62 (45.3%)	75 (54.7%)	0.66 (0.31, 1.38)	0.60 (0.26, 1.39)
Work shift	Fixed	60 (34.3%)	115 (65.7%)	1.00	1.00
	Rotating	138 (57.7%)	101 (42.3%)	2.61 (1.74, 3.92)*	2.89 (1.87, 4.45)**
Hours worked per day	≤8 h	127 (50.8%)	123 (49.2%)	1.00	1.00
	>8 h	71 (43.3%)	93 (56.7%)	0.73 (0.49, 1.09)*	0.75 (0.48, 1.16)

CI = confidence interval, COR = crude odds ratio, AOR = adjusted odds ratio.

* *p* < 0.25;

** *p* < 0.05.

## 4. Discussion

The prevalence of occupational stress among nurses was found to be 47.8% in this study, which is higher than the studies conducted in Isfahan, Iran, which found that the prevalence of stress was 34.9% (32) and Addis Ababa, Ethiopia, which found that the prevalence of occupational stress among nurses was 37.8% (33). The difference could be a result of the different tools used and the sample size, but another explanation could be that Isfahan, Iran, had stronger occupational health and safety practices implemented.

However, the results of this study are less significant than those of earlier research done in Delhi, which found that 87.4% of nurses experienced job-related stress, and in Jima Zone South West Ethiopia, which found that the average level of job-related stress was 58.46 ± 12.62 (15, 34). When compared to the study conducted in Jima, this may be related to sample size, however in Delhi, the discrepancy may be caused by study tools and the study location.

According to this study, the four main sources of stress for nurses are “death and dying,” “uncertainty regarding patient treatment,” “conflict with physician,” and “work load.” The biggest source of stress, in respondent’s opinions, was death and dying. The current study found that dealing with death and dying situations is a significant source of stress, which is consistent with studies conducted in Sudan, where dealing with death and dying situations had the highest mean scores of ENSS, mean = 2.23, Standard deviation = 0.56, and in Jima, where the highest stressful condition that nurses rated as always stressful was the death and dying of a patient with 62.94%, followed by uncertainty regarding patient treatment with 57.72% (15, 35). The study participants’ cultural and humanitarian sympathy may be the cause of their emotional problems with relation to the patient’s death or dying.

Uncertainty regarding the treatment subscale was the second cause of work-related stress in this study. Similar findings were found in a research conducted among 135 ICU nurses at the Children’s University Hospital at El-Shatby (Egypt), which demonstrated “death and dying” followed, by uncertainty concerning the treatment (36). This could be as a result of a lack of knowledge, experience, or expertise in dealing with unforeseen and challenging issues.

The third source of stress had a mean score of 2.47 and was conflict with physician subscale. Conflict with physician was identified as a source of work-related stress in studies conducted in Spain, which is consistent with this finding (37). This may be due to a lack of relationships, communication, and cooperation.

Many of socio-demographic and workplace factors in this study had no statistically significant relationships with overall occupational stress. This may be the tool’s strongest attribute. In multivariate logistic regression, the only significant predictors of occupational stress were having children and working a shift.

According to this study, there is a significant association between having children and workplace stress. Nurses who did not have children reported being 54% less stressed than those who did (AOR: 0.46, 95% CI: 0.22, 0.96). This may be because raising children increases the workload for these nurses. This study is in line with one done in Kampala, Uganda, which found that nurses in Ugandan hospitals deal with a fair amount of occupational stress. Additionally, the findings revealed that nurses without children experienced much less occupational stress than those with children (38).

A significant relationship between work shift and stress at work was also discovered in this study; rotating shift nurses reported higher levels of stress than fixed shift nurses (AOR = 2.89, 95% CI: 1.87, 4.45). This result was in line with a study conducted in Addis Ababa, which found that nurses working rotational shifts experienced higher levels of stress than those working fixed shifts (39). Additionally, this result was consistent with study conducted in Egypt that found the work shift was the strongest predictor of nurses’ stress (36) and that revealed nurses working the rotating shift were more stressed than those who worked the morning shift (40).

## 5. Limitations of the study

Since stress is mainly subjective and psychological, the qualitative approach would provide rich and meaningful information about the nurses’ experiences with stress and related concepts.

## 6. Conclusion

In this study, about half of the nurses reported experiencing occupational stress. The presence of children and respondents’ work shifts were personal characteristics that were significantly linked to job stress. The biggest drivers of work-related stress for nurses were death and dying, treatment uncertainty, conflict with physician, and problem with peers.

According to these findings, Ministry of Health and Addis Ababa Health Bureau and Nursing stake holders should collaborate to design stress management programs for nurses that include the proactive identification and evaluation of stressors in work areas. All hospitals and nursing administrators must take responsibility for the health and well-being of their staff by reducing stressful situations. They should reschedule shifts and recruit adequate nurses, decreasing workloads. Furthermore, support systems such as counseling services and self-help groups should be made available to nurses.

## Data availability statement

The original contributions presented in the study are included in the article/supplementary material, further inquiries can be directed to the corresponding author.

## Ethics statement

Ethical clearance was obtained from Addis Ababa Health Bureau before data collection. Supportive letter was obtained from Kotebe University of Education, Menelik II College of Medical and Health Science. Oral consent was obtained from each study participants during data collection. Right was given to study participants to refuse, stop, or withdraw from the interview at any time. Confidentiality was maintained throughout the study.

## Author contributions

EW wrote the proposal, participated in data collection, analyzed the data, wrote the manuscript, and approved the manuscript for publication. ZW reviewed and approved the manuscript. All authors contributed to the article and approved the submitted version.

## Conflict of interest

The authors declare that the research was conducted in the absence of any commercial or financial relationships that could be construed as a potential conflict of interest.

## Publisher’s note

All claims expressed in this article are solely those of the authors and do not necessarily represent those of their affiliated organizations, or those of the publisher, the editors and the reviewers. Any product that may be evaluated in this article, or claim that may be made by its manufacturer, is not guaranteed or endorsed by the publisher.

## Abbreviations

AOR: Adjusted odd ratio
CI: Confidence Interval
COR: Crude odd ratio
COVID-19: Coronavirus disease 2019
ENSS: Expanded nursing stress scale
HSDP: Health sector development program
ICU: Intensive care unit
NIOSH: National institute for occupational safety and health
OR: Odd ratio
SPSS: Statistical package of social sciences
SD: Standard deviation
WRS: Work related stress
